# A case of a giant cell myocarditis that developed massive left ventricular thrombus during percutaneous cardiopulmonary support

**DOI:** 10.1186/s40981-016-0067-0

**Published:** 2016-11-29

**Authors:** Yusuke Takei, Yutaka Ejima, Hiroaki Toyama, Kana Takei, Takahisa Ota, Masanori Yamauchi

**Affiliations:** 1 Department of Anesthesiology, Tohoku University Hospital, 1-1 Seiryomachi, Aoba-ku, Sendai, 980-8574 Japan; 2 Division of Surgical Center and Supply, Sterilization, Tohoku University Hospital, 1-1 Seiryomachi, Aoba-ku, Sendai, 980-8574 Japan; 3 Anesthesiology and Perioperative Medicine, Tohoku University School of Medicine, 2-1 Seiryomachi, Aoba-ku, Sendai, 980-8575 Japan

**Keywords:** Giant cell myocarditis, Ventricular thrombus, Percutaneous cardiopulmonary support system

## Abstract

**Background:**

Giant cell myocarditis, characterized by infiltration of multinucleated giant cells in the myocardium, is a rare type of myocarditis. It often progresses rapidly into fulminant heart failure and indicates a poor prognosis. When a patient with giant cell myocarditis develops into severe myocarditis presenting with a cardiogenic shock, we should use a percutaneous cardiopulmonary support (PCPS), which could occur complications. We experienced a patient with giant cell myocarditis, who developed left ventricular thrombus formations during the circulation support therapy with PCPS for cardiogenic shock.

**Case presentation:**

A 60-year-old man who developed a cardiogenic shock was transferred to our hospital. After the admission, inotropic agents were increased and an intra-aortic balloon pumping was started. But these therapies did not improve his hemodynamic status. He was placed PCPS. Then, he underwent endomyocardial biopsy and was diagnosed with giant cell myocarditis. On the next morning, he developed complete atrioventricular block, and subsequently, thrombus formations occurred in his left ventricular outlet tract and Valsalva sinus despite an anticoagulant therapy. Thereafter, we intensified the anticoagulant therapy to prevent further thrombus formation, but he developed an intracranial hemorrhage. He did not recover from heart failure and died 16 days after the admission.

**Conclusions:**

We present a patient with giant cell myocarditis who developed widespread thrombosis in the left ventricle during the circulatory support with PCPS, despite anticoagulant therapy. In this case, decreased left myocardial contractility caused by giant cell myocarditis and increased left ventricular afterload by the retrograde perfusion from the PCPS induced the thrombotic tendency and congestion in the left ventricle. In addition, he developed complete atrioventricular block, which reduced the left ventricular ejection and enhanced the thrombus formation. Because patients with giant cell myocarditis have a low probability of spontaneous recovery, heart transplantation or ventricular assist device implantation may be required for circulatory support. We should establish mechanical circulatory support rapidly to improve the prognosis of patients with giant cell myocarditis. Moreover, a ventricular assist device, which can prevent both ventricular congestion and retrograde blood flow, might be suitable to prevent complications as this case.

## Background

Giant cell myocarditis, characterized by infiltration of multinucleated giant cells in the myocardium, is a rare type of myocarditis. Fulminant myocarditis can occur during its clinical course. When it occurs, prognosis is extremely poor due to acute heart failure [1–4]. Herein, we report a patient with giant cell myocarditis who developed massive left ventricular thrombus during the circulatory support with percutaneous cardiopulmonary support (PCPS) for the treatment of progressive heart failure.

## Case presentation

**Fig. 1:**
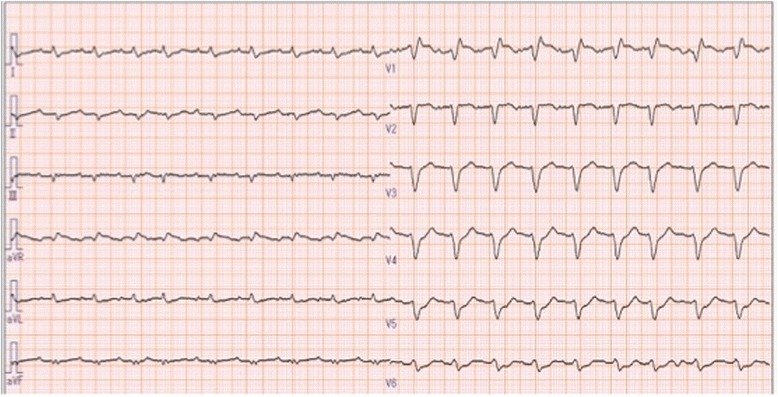
The 12-lead electrocardiogram of the patient upon admission to the intensive care unit. Sinus rhythm, low-voltage R-wave, and intraventricular conduction disturbance in all leads were seen

A 60-year-old man noted cold-like symptoms. After a month, he was visited to a local hospital and was suspected with myocarditis because his blood examination showed high inflammatory reactions and his echocardiogram showed severe cardiac dysfunction. He was started on intravenous continuous infusions of dobutamine (5 μg/kg/min) and dopamine (5 μg/kg/min) and transferred to our hospital. Upon admission to the intensive care unit, his consciousness, heart rate, blood pressure, arterial oxygen saturation level (5 L/min of supplemental oxygen via facemask), and body temperature were clear, 105 bpm, 105/66 mmHg, 100%, and 38.2 °C, respectively. His chest radiograph and chest computed tomography showed pulmonary congestion and bilateral pleural effusion without cardiomegaly, and the electrocardiogram showed low voltage of R-wave and intraventricular conduction disturbance in all leads (Fig. 1). His laboratory examination showed inflammatory reaction, elevated levels of cardiac enzyme, and serum brain natriuretic peptide but almost normal range of coagulation (Table 1). His transthoracic echocardiogram showed a significant decrease in the myocardial contractility of the both ventricles but did not show the enlargement of both ventricles and a thrombus formation in all ventricles. Right cardiac catheterization, under the continuous infusions of dopamine (5 μg/kg/min) and dobutamine (5 μg/kg/min), showed that cardiac output (CO), pulmonary arterial pressure (PAP), pulmonary arterial wedge pressure, and mixed venous oxygenation saturation (S_v_O_2_) were 3.60 L/min, 37/18(26) mmHg, 26 mmHg, and 40%, respectively, which indicated cardiogenic shock and post-capillary pulmonary hypertension (Table 2). Coronary angiography showed no abnormal findings. Because he was diagnosed with myocarditis and refractory to inotropic agents, he was inserted with intra-aortic balloon pumping (IABP). Despite 6 h of IABP support, his hemodynamic status progressively deteriorated; hence, we decided to start the mechanical ventilation and mechanical circulatory support by a PCPS on him. We administered 1 mg of midazolam, 0.2 mg of fentanyl, and 50 mg of rocuronium, performed endotracheal intubation, and started the airway pressure release ventilation with fraction of inspired oxygen (F_I_O_2_) of 0.6, the high continuous airway pressure (CPAP) of 20 cmH_2_O, duration of high CPAP of 9.5 s, low CPAP of 0 cmH_2_O, and duration of low CPAP of 0.5 s in order to avoid the lung collapse. In this setting, his minute ventilation volume was 2.4 L (approximately 400 mL of tidal volume and 6/min of respiratory rate). After the initiation of the mechanical ventilation, his arterial blood gas analysis from the right radial artery showed a pH of 7.499, arterial oxygen tension (PaO_2_) of 153 mmHg, and arterial carbon dioxide (CO_2_) tension (PaCO_2_) of 34.8 mmHg. And we started continuous infusion of propofol (1–2.5 mg/kg/h) and fentanyl (40 μg/h) for sedation. Then, we administered heparin (7000 IU) as an intravenous bolus, and his activated clotting time (ACT) was prolonged to 198 s. We placed a venous cannula into the right atrium via the common right femoral vein and an arterial cannula into the left femoral artery and started the PCPS.

**Table 1:** The blood examination on admission and 1 and 10 days after the admission

	Admission	1 day after the admission (after PCPS placement)	10 days after the admission (after intensive anticoagulant therapy)
White blood cell counts (/μL)	9200	9100	12,400
Hemoglobin (g/dL)	10.2	10.6	10.7
Platelet counts (×10^4^/μL)	38.2	29.3	27.6
Total bilirubin (mg/dL)	0.8	0.7	1.5
Aspartate transaminase (IU/L)	401	431	194
Alanine aminotransferase (IU/L)	403	367	236
Lactate dehydrogenase (IU/L)	1081	926	932
Blood urea nitrogen (mg/dL)	18	17	15
Creatinine (mg/dL)	1.18	1.01	0.94
C-reactive protein (mg/dL)	13.2	10.9	17.1
Creatine kinase (IU/L)	1194	776	871
Creatine kinase-MB (IU/L)	32	32	39
Troponin T (ng/dL)	5.19	–	–
Brain natriuretic peptide (pg/mL)	3035	2266	–
Lactate (mmol/L)	1.20	1.12	0.97
BE (mmol/L)	−0.2	−0.1	−1.3
International normalized ratio of prothrombin time	1.14	1.16	1.21
Activated partial thromboplastin time (s)	30.0	38.7	118.9
Activated clotting time (s)	140	198	273

Almost the normal ranges of coagulation test were found on admission. The marked prolonged activated partial thromboplastin time and activated clotting time were found after the intensive anticoagulation therapy for the intraventricular thrombus

**Table 2:** Hemodynamic status of the patient on admission, before and after the initiation of PCPS, after the onset of complete atrioventricular block, and after ventricular pacing

	Admission	Before initiation of PCPS	Initiation of PCPS	Onset of complete AVB	After ventricular pacing
Respiratory support	Face mask, 5 L/min of oxygen	Face mask, 6 L/min of oxygen	APRV, F_I_O_2_ = 0.6, *P*_high_ = 20 cm H_2_O, *T*_high_ = 9.5 s, *P*_low_ = 0 cm H_2_O, *T*_low_ = 0.5 s	Same as on the left	APRV, F_I_O_2_ = 0.6, *P*_high_ = 15 cm H_2_O, *T*_high_ = 5.5 s, *P*_low_ = 0 cm H_2_O, *T*_low_ = 0.5 s
pH	7.531	7.532	7.449	7.272	7.554
SaO_2_ (%)	95.1	97.0	98.5	95.7	98.5
PaO_2_ (mmHg)	75.4	97.0	153.0	100.8	158.0
PaCO_2_ (mmHg)	25.5	28.1	34.8	62.5	27.3
EtCO_2_ (mmHg)	–	–	21	41	17
BE (mmol/L)	−0.2	1.7	−0.1	0.3	1.9
Lac (mmol/L)	1.2	1.3	1.12	1.03	1.28
ABP (mmHg)	110/55	112/42	97/41	70/40	136/37
PAP (mmHg)	37/18 (26)	28/11	19/10	60/20	18/13
PAWP (mmHg)	25	–	12	30	–
RAP (mmHg)	18	18	12	18	8
CO (L/min)	3.60	3.80	2.3	<<1.0	1.3
Output of PCPS (L/min)	–	–	3.0	3.0	3.5
The sum of PCPS blood flow and his own cardiac output (L/min)	3.60	3.80	5.3	3.0	4.8
Total gas flow of oxygenator (L/min)	–	–	1.0	1.0	4.0
SvO_2_ (%)	40	39.7	66	55	69

The cardiogenic shock was improved by the PCPS support. After the onset of complete AV block, significant decrease of CO, pulmonary congestion, and increased PaCO_2_ were observed

*PCPS* percutaneous cardiopulmonary support, *AVB* atrioventricular block, *APRV* airway pressure release ventilation, *F*_*I*_ *O*_*2*_ fraction of inspired oxygen, *P*_*high*_ high continuous positive airway pressure, *T*_*high*_ duration of *P*_high_, *P*_*low*_ low continuous positive airway pressure, *T*_*low*_ duration of *P*_low_, *EtCO*_*2*_ end-tidal carbon dioxide tension, *PaCO*_*2*_ arterial carbon dioxide tension, *ABP* arterial blood pressure, *PAP* pulmonary artery pressure, *PAWP* pulmonary artery wedge pressure, *RAP* right atrial pressure, *CO* cardiac output, *SvO*_*2*_ mixed venous oxygenation saturation

At the initiation of PCPS, the target value of PCPS blood flow was set at 3.0 L/min (rotation speed of 3000 rpm), and the F_I_O_2_ and total gas flow of the membrane oxygenator were set at 0.6 and 1 L/min, respectively.

And the intravenous continuous infusion of heparin (10,000 IU/day) was started in order to maintain his ACT within the range of 150 to 200 s. After the PCPS blood flow reached to 3.0 L/min, the SvO_2_ increased to 66% and the total blood flow, which was the sum of PCPS blood flow and his own cardiac output, increased from 3.0 to 5.3 L/min (Table 2).

**Fig. 2:**
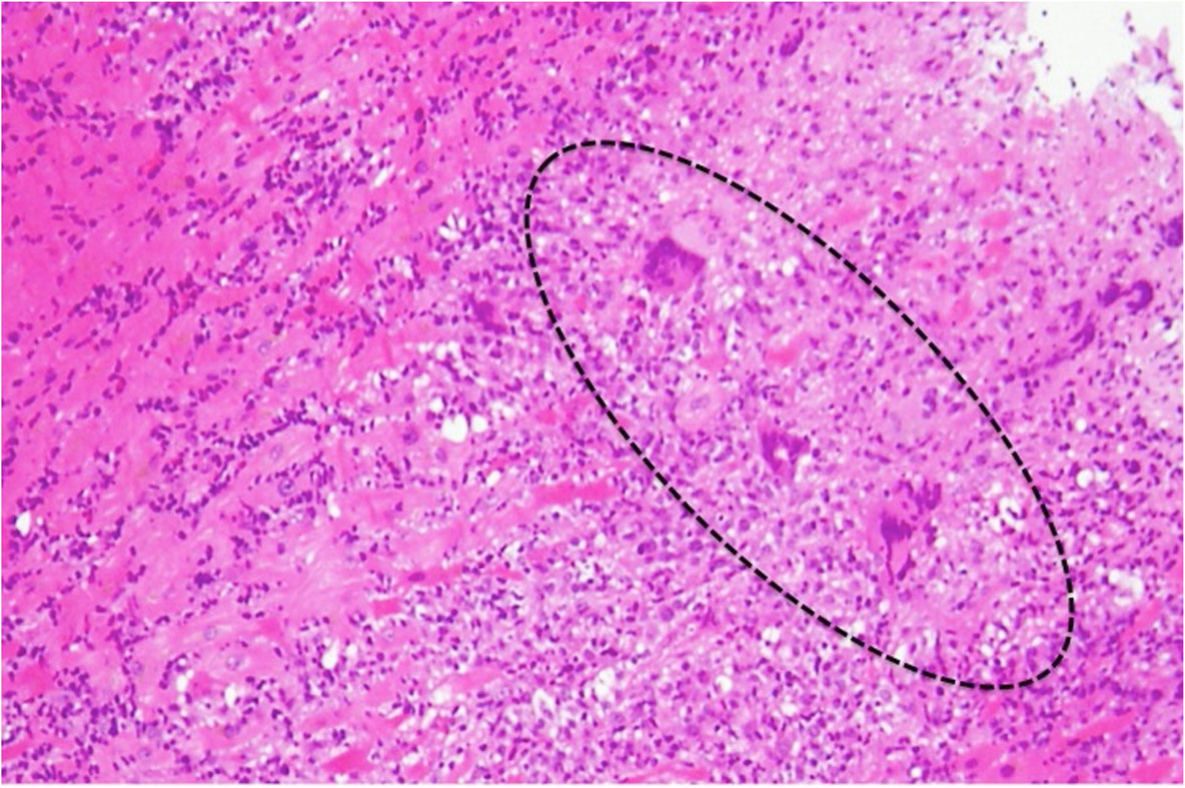
Microscopic examination from the endomyocardial biopsy of the right ventricle. Characteristic infiltrations of multinucleated giant cells in the myocardium (*open circle*) were seen

Subsequently, we performed endomyocardial biopsy (EMB) followed by rapid microscopic examination, which showed the characteristic infiltration of multinucleated giant cells in the myocardium. He was eventually diagnosed with giant cell myocarditis (Fig. 2).

**Fig. 3:**
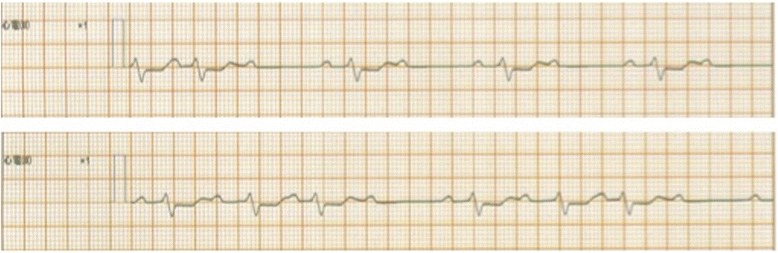
Electrocardiogram strips on the next morning. Complete atrioventricular block was seen

**Fig. 4:**
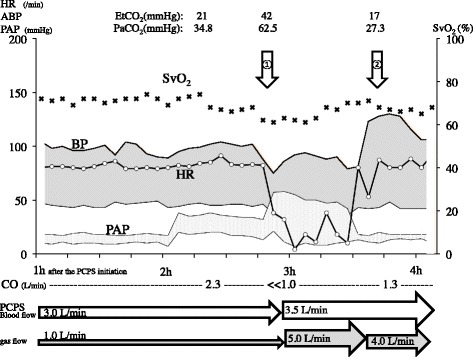
The change in the hemodynamic status around the onset of the atrioventricular block. *HR* heart rate, *ABP* arterial blood pressure, *PAP* pulmonary artery pressure, *P*_*E*_ *tCO*_*2*_ end-tidal carbon dioxide tension, *PaCO*_*2*_ arterial carbon dioxide tension, *SvO*_*2*_ mixed venous oxygenation saturation, *PCPS* percutaneous cardiopulmonary support, CO cardiac output. ① The onset of the complete atrioventricular block. ② The start of the ventricular pacing. After the onset of the complete atrioventricular block, the decrease of BP and the increase of PAP and PaCO_2_ continued until the point when the blood flow and the gas flow of the PCPS were augmented. The hemodynamics of the patient improved along with the recovery of the ventricular contraction by the ventricular pacing

**Fig. 5:**
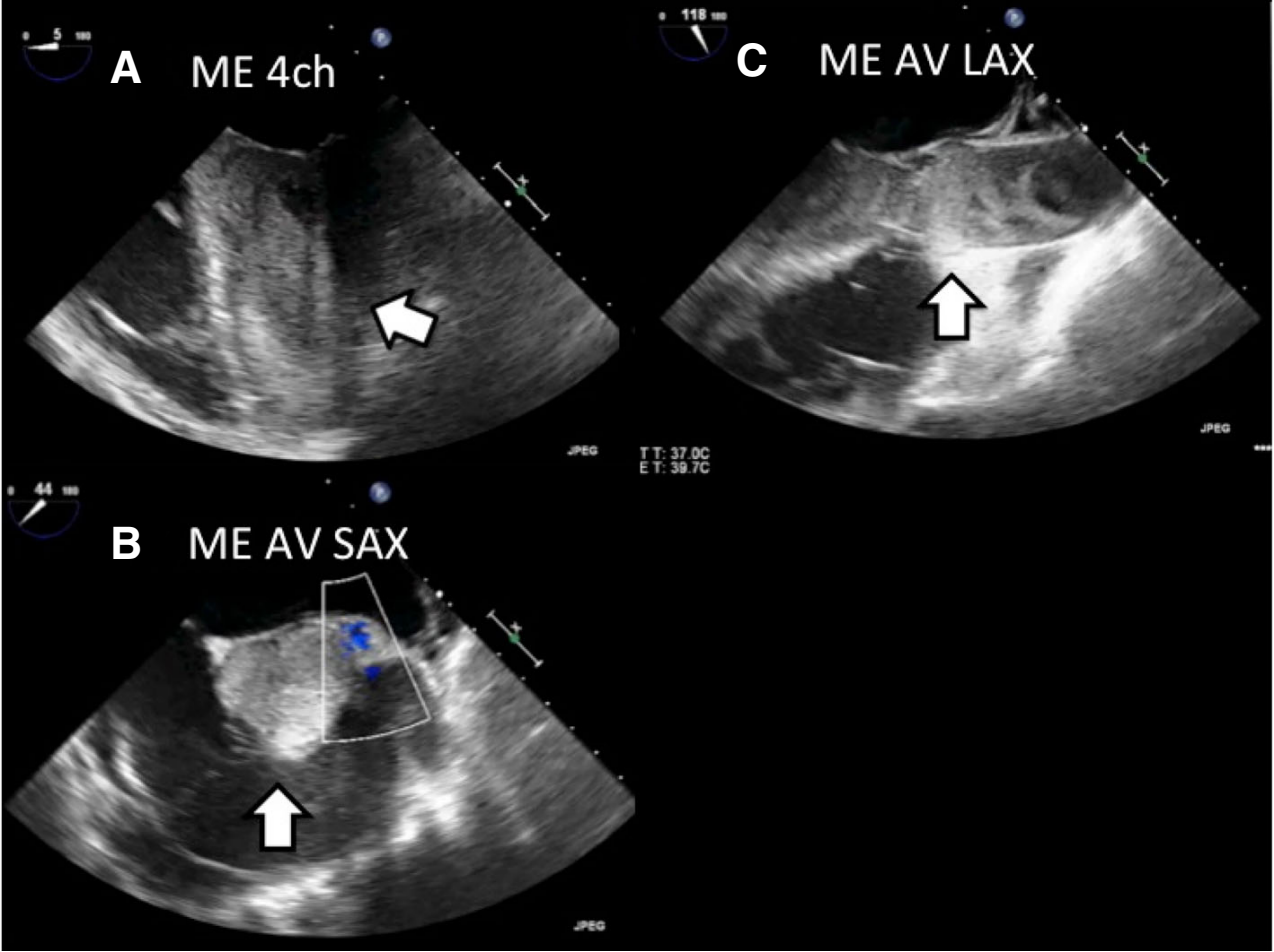
Transesophageal echocardiography (TEE) after the onset of atrioventricular block. **a** Mid-esophageal 4-chamber (ME 4ch) view showed thrombus formations and congestion in the left ventricle. **b** Mid-esophageal aortic valve short-axis (ME AV SAX) view showed thrombus formations in the Valsalva sinus and aortic valve closure. **c** Mid-esophageal long-axis (ME AV LAX) view showed thrombus formations in the left ventricular tract and the Valsalva sinus

On the next morning, he developed complete atrioventricular block (Fig. 3), subsequent decreased blood pressure from 100/50 to 70/40 mmHg, and increased pulmonary arterial pressure from 35/15 to 60/20 mmHg. In addition, sudden elevation of PaCO_2_ from 31 to 62.5 mmHg was also observed despite the preservation of both the steady PCPS output and the mechanical ventilation setting (Fig. 4 and Table 2). Hence, we performed transesophageal echocardiography (TEE), which indicated widespread thrombus formations and marked congestion in his left ventricular outlet tract and Valsalva sinus and closure of the aortic valve; however, there is no thrombus in his right ventricle (Fig. 5). Then, we checked ACT, which was 168 s, and performed an additional test for heparin-induced thrombocytopenia (HIT) antibody, which we learned of the negative result later.

We increased the PCPS blood flow up to 3.5 L/min and total gas flow up to 4.0 L/min, which lead to success of CO_2_ excretion, but his hemodynamic status remained unstable. After that, we started temporary cardiac pacing to treat complete atrioventricular block, which restored his left ventricular contractility, opened the aortic valve, and subsequently washed out the thrombus.

Thereafter, for the purpose of preventing further thrombus formation, anticoagulant therapy was intensified (20,000 IU/day of heparin) to maintain ACT within the range of 200 to 240 s. Ten days after the admission, his ACT was markedly prolonged up to 273 s (Table 2) and he presented with an anisocoria. Therefore, we performed a computed tomography (CT) scan of the brain, which revealed the intracranial hemorrhage. He did not recover from the heart failure and died 16 days after the admission.

### Discussion

Giant cell myocarditis is characterized by infiltration of multinucleated giant cells in the myocardium and distinguished from cardiac sarcoidosis by the absence of non-caseous necrosis. Cardiac sarcoidosis is generally associated with extracardiac lesions and progresses slowly, whereas giant cell myocarditis develops and progresses to heart failure rapidly, which results in poor prognosis [1–3]. An accurate data about the morbidity of giant cell myocarditis has not been shown because the pathological diagnosis of the disease is difficult. However, approximately 100 cases were reported despite the rarity of this myocarditis type [4]. Mechanical circulatory support, such as IABP and/or PCPS as well as inotropes, is required for the treatment of acute giant cell myocarditis because the disease often progresses to fulminant myocarditis. Some reports showed the efficacy of immunosuppressive therapy for giant cell myocarditis, which suggested a relationship between giant cell myocarditis and autoimmune reaction [5–7]. In addition, a multicenter study of immunosuppressive therapy for giant cell myocarditis showed the prolonged median transplant-free survival from 3 to 12.3 months [1]. However, some studies also showed that few patients recovered from heart failure and weaned from mechanical circulatory support [7–9]. The patients of that study eventually required heart transplantation or circulatory support, such as ventricular assist device (VAD) for destination therapy. In Japan, VAD is a practical option for the rescue treatment of chronic giant cell myocarditis because only 40 heart transplantations are performed per year, and the mean waiting period for transplantation is 636 days. We need to transfer the patients to the hospital capable for left VAD (LVAD) surgery, which is limited in Japan, if needed for LVAD placement.

We could not save this patient because of the disease progression and the complications, including the thrombosis due to the congestion of the left ventricle and intracranial hemorrhage due to the intensive anticoagulant therapy to prevent thrombosis.

**Fig. 6:**
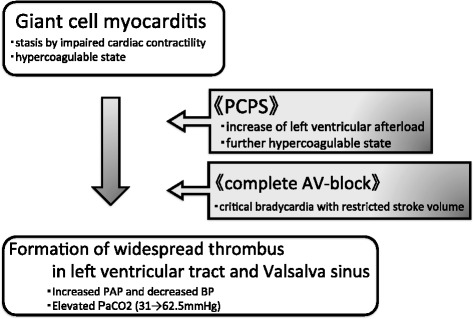
The mechanism of thrombus formation in this case. *PCPS* percutaneous cardiopulmonary support, *AV-block* atrioventricular block, *BP* systemic blood pressure, *PAP* pulmonary artery pressure, *PaCO*_*2*_ arterial carbon dioxide tension. Giant cell myocarditis induced the left ventricular distension and subsequent thrombus formation tendency. The retrograde perfusion by the PCPS and the complete atrioventricular block further enhanced thrombus formation tendency. As a result, widespread thrombi were formed in the left ventricular tract and the Valsalva sinus

Myocarditis alone rarely accompanies ventricular thrombosis unlike dilated cardiomyopathy. Left ventricular distension strongly contributes to intraventricular thrombosis. Therefore, the cornerstone in prevention of intraventricular thrombosis is systemic anticoagulant therapy and prevention of left ventricular distension. In this case, thrombotic tendency was induced by the left ventricular congestion due to decreased left myocardial contractility. Regardless of promoting the left ventricular ejection by inotropic agents and IABP, adequate cardiac output was not obtained, which resulted in the placement of PCPS. However, peripheral PCPS perfused the aortic root retrogradely, which caused the increase of left ventricular afterload, the subsequent distension of the left ventricle, and the further increase of thrombotic tendency and congestion at the left ventricular outlet tract. Approximately 15% of patients with giant cell myocarditis are associated with atrioventricular block [1, 4]. In this case, the complete atrioventricular block induced a critical decrease of the left ventricular ejection, which resulted in the further left ventricle distension, which might trigger the thrombosis (Fig. 6).

Before the onset of the atrioventricular block, the patient had 2.3 L/min of the pulmonary circulation with 2.4 L/min of minute volume of ventilation and 3.0 L/min of the PCPS blood flow with 1 L/min of gas flow of the artificial lung. In this condition, the main pathway of his CO_2_ elimination was thought to be the pulmonary circulation. After the onset of the atrioventricular block, the pulmonary circulation decreased to far less than 1.0 L/min because of the loss of the cardiac output while 3.0 L/min of the PCPS blood flow was maintained. Hence, the total lung blood flow decreased to approximately 3.0 L/min, and the artificial lung ought to excrete almost whole CO_2_. However, the gas flow of the artificial lung remained 1.0 L/min, which might be too little to excrete sufficient amount of the CO_2_. Thereafter, the PaCO_2_ of the patient rose from 34.8 to 62.5 mmHg.

To our knowledge, no guideline is available for treatment of intraventricular thrombosis in PCPS patients. The treatment choices are thrombectomy and intensive anticoagulant therapy [10–12]. Because of the unstable circulatory status of the patient, intensive systemic anticoagulant therapy and temporary cardiac pacing, which could recover the ventricular contractility and ejection, were the possible choices. Therefore, we started temporary cardiac pacing, which recovered the left ventricular contractility, opened the aortic valve, and washed out the thrombi. Additionally, the efficacy of local thrombolysis in intraventricular thrombosis was recently reported. This technique might be effective in this case [13].

The washed out thrombi formed at the onset of the complete atrioventricular block could cause cerebral infarctions, and the intensified coagulation therapy might deteriorate the cerebral infarctions into the intracranial hemorrhage. At the onset of complete atrioventricular block, we did not perform a CT scan of the brain or awaken him from the deep sedation due to his unstable circulatory status. But 10 days after the admission, we performed a CT scan of the brain at the risk of circulatory failure because the patient presented with an anisocoria. However, we could not identify the true pathogenesis of the intracranial hemorrhage.

Different types of myocarditis show different clinical courses and prognoses and require different treatments. Therefore, EMB, identifying the histological type of myocarditis, should be performed as the patient’s condition permits. In this case, multidrug immunosuppressive therapy was started after the histological diagnosis of giant cell myocarditis. However, as previously described, heart transplantation or VAD may be required for a definitive therapy within months or years. LVAD, withdrawing the blood from the left ventricular apex and returning the blood to the ascending aorta, can establish physiological perfusion and reduce left ventricular volume, whereas peripheral PCPS perfuses aorta retrogradely, increases left ventricular afterload, and might induce left ventricular distension. In addition, LVAD can cause less bleeding complications than PCPS because LVAD requires weaker anticoagulation than PCPS [14]. If we could place an LVAD on the patient at the diagnosis of giant cell myocarditis, complications such as thrombosis and bleeding might be prevented, which might result in smooth bridge to heart transplantation. To improve the prognosis of myocarditis, full knowledge of the clinical course of myocarditis, precise diagnosis, transfer to an advanced medical center where LVAD placement can be performed and rapid establishment of adequate mechanical circulatory support should be required.

## Conclusions

We present a patient with giant cell myocarditis who developed widespread thrombosis in the left ventricle during PCPS support. Retrograde perfusion by PCPS and complete atrioventricular block promoted left ventricular distension, reduced left ventricular ejection, and caused widespread thrombosis. In the circulation management of patients with giant cell myocarditis, we should rapidly establish physiological mechanical circulatory support.

## Abbreviations

ACT: Activated clotting time
CO: Cardiac output
CO_2_: Carbon dioxide
CPAP: Continuous positive airway pressure
CT: Computed tomography
EMB: Endomyocardial biopsy
F_I_O_2_: Fraction of inspired oxygen
HIT: Heparin-induced thrombocytopenia
IABP: Intra-aortic balloon pumping
LVAD: Left ventricular assist device
PaCO_2_: Arterial carbon dioxide tension
PaO_2_: Arterial oxygen tension
PAP: Pulmonary arterial pressure
PCPS: Percutaneous cardiopulmonary support
SvO_2_: Mixed venous oxygenation saturation
TEE: Transesophageal echocardiography
VAD: Ventricular assist device
